# Oceanic vortex mergers are not isolated but influenced by the *β*-effect and surrounding eddies

**DOI:** 10.1038/s41598-020-59800-y

**Published:** 2020-02-19

**Authors:** Charly de Marez, Xavier Carton, Pierre L’Hégaret, Thomas Meunier, Alexandre Stegner, Briac Le Vu, Mathieu Morvan

**Affiliations:** 1 Univ. Brest, Laboratoire d’Océanographie Physique et Spatiale (LOPS), Plouzané, France; 2 CICESE, Ensenada, B.C. Mexico; 3 Laboratoire de Météorologie Dynamique, CNRS Ecole Polytechnique, Palaiseau, France

**Keywords:** Physical oceanography, Fluid dynamics

## Abstract

Oceanic vortices are ubiquitous in the ocean. They dominate the sub-inertial energy spectrum, and their dynamics is key for the evolution of the water column properties. The merger of two like-signed coherent vortices, which ultimately results in the formation of a larger vortex, provides an efficient mechanism for the lateral mixing of water masses in the ocean. Understanding the conditions of such interaction in the ocean is thus essential. Here, we use a merger detection algorithm to draw a global picture of this process in the ocean. We show that vortex mergers are not isolated, contrary to the hypothesis made in most earlier studies. Paradoxically, the merging distance is well reproduced by isolated vortex merger numerical simulations, but it is imperative to consider both the *β*-effect and the presence of neighbouring eddies to fully understand the physics of oceanic vortex merger.

## Introduction

Oceanic vortices, named *eddies*, impact biological activities^1^, tracer transport^2^, and properties of the water column^3^. It has become clear that the mesoscale (10–100 km) eddy field, is at least as energetic as the large scale circulation^2^, essential for the air-sea interactions^4^, and thus for the evolution of climate^5^. Eddy-eddy interactions therefore play a central role in the evolution of the ocean/atmosphere physics and biology. In particular, *vortex merger* —the physical process in which two like-signed coherent vortices collapse, ultimately resulting in a larger vortex— is of key importance for the distribution and the transfers of energy across scales in the ocean^6–11^. Since the 80’s, an important effort has been undertaken to understand the physics of oceanic vortex merger, including *in situ*^12^, laboratory^13,14^, and numerical^15^ observations, associated with intense theoretical debates^16–18^. Most fundamental studies addressed the ’*isolated* vortex merger’ problem, omitting the influence of neighbouring eddies, large scale currents, or boundary, topographic, and planetary effects^19–25^. Efforts to include more physical effects in studies of vortex merger^26–29^ were often impaired by the lack of general observations in the global ocean, or by the complexity of the resulting dynamical system. Despite recent trials^30^ and pointwise observations^12,31–33^, a global description of vortex merger in the ocean is lacking.

**Figure 1:**
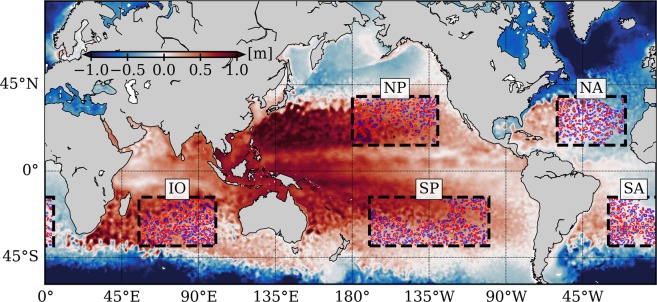
Definition of the five study areas. The color map represents a snapshot of Sea Surface Height on 01/05/2000 from the HYCOM dataset. Eddies detected on this date are presented by the red and blue contours (for cyclonic and anticyclonic eddies respectively). NP, SP, NA, SA, and IO are abbreviations for North Pacific, South Pacific, North Atlantic, South Atlantic, and Indian Ocean.

In this paper, we present an analysis of merging events in the global ocean, using a global 1/12° re-analysis dataset. This study was conducted over a five-year period, in five domains in the middle of each major oceanic basins —these domains *a priori* respect the hypothesis of isolated vortex merger studies: they are far away from the coastlines, topographic features, and strong currents, and are located at mid-latitudes (see Fig. 1). From 5,867 detected merging events, we infer the actual characteristics of mesoscale eddies that merge, and test the isolated vortex merger problem hypotheses. Further, we compare this analysis with the outcome of 1,600 idealized numerical simulations. The latter rely on a 3D primitive equations model, both on the *f*-plane and on the *β*-plane, using a realistic open ocean parameterization (*e.g*. a realistic stratification, and numerical parameters used in regional numerical simulations^34,35^). To allow for a direct comparison, we use the same merging event detection algorithm in both datasets.

Here, we show that in the five study areas, vortex mergers are not isolated; they are influenced by neighbouring eddies as well as by the *β*-effect. Paradoxically, the merging process in the ocean is well represented by very simple simulations of Gaussian eddies a thousand meters deep on an *f*-plane, at least as far as the critical distance of merging is concerned. The necessary inclusion of *β*-effect for realism leads to a discrepancy with the global analysis: the advection of eddies by planetary Rossby waves weaken the critical distance of merging. Thus, another physical effect is at work in the real ocean: the ubiquitous density of the mesoscale eddy field, which prevent the eddies to drift away during the merger. These findings have important implications for the future understanding of eddy-eddy interactions.

## Results

### Vortex merging analysis in the ocean: are interacting vortex pairs isolated from the surrounding ?

**Figure 2:**
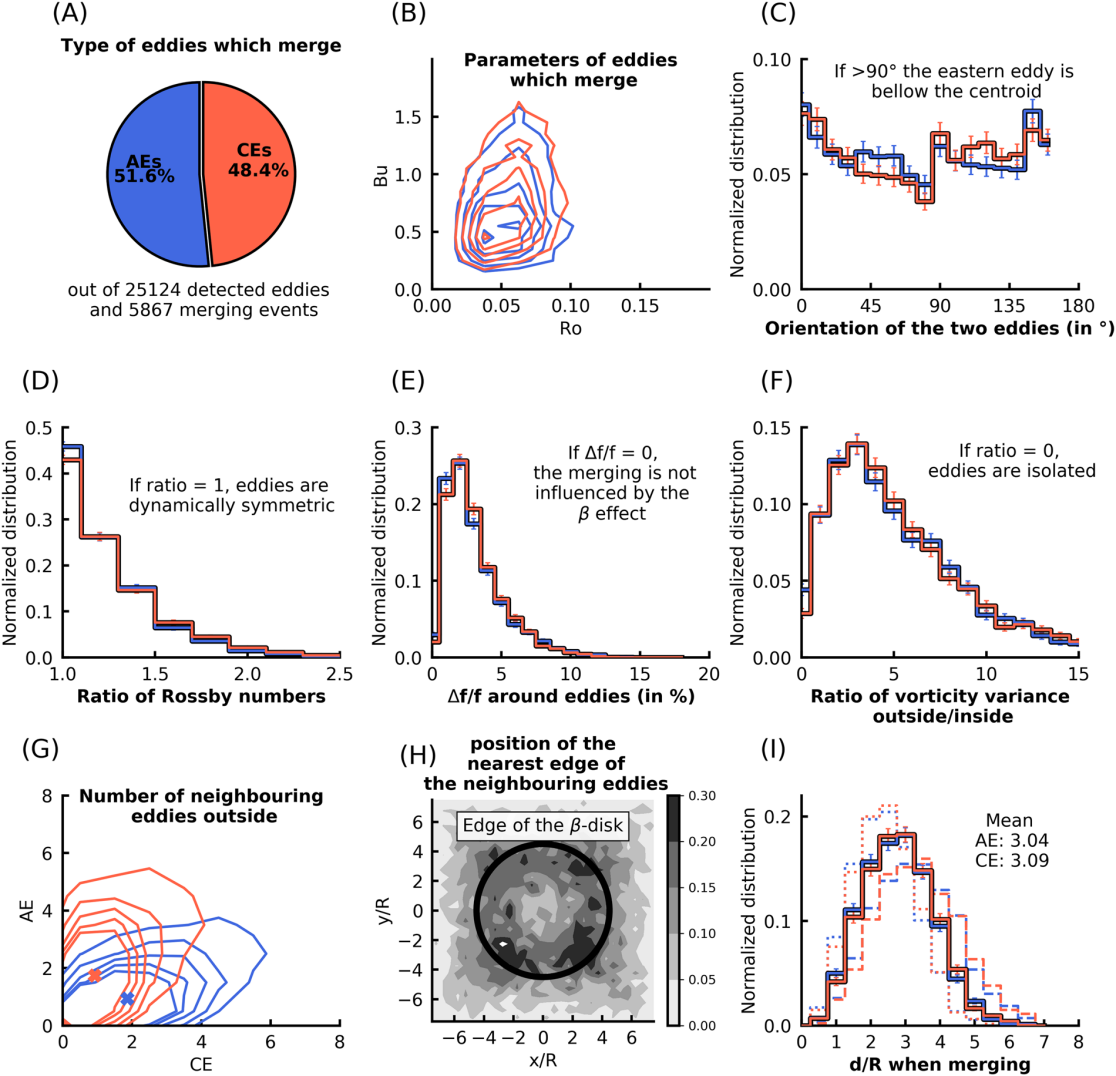
Characteristics of the merging events from the HYCOM dataset. In each panel, blue and red indicate Anticyclonic Eddies (AEs) and Cyclonic Eddies (CEs) respectively. (**A**) Type of eddies which merge; the distribution is normalized by the number of eddies per type (we take into account the fact that more AEs are detected). (**B**) *Ro* and *Bu* distribution, contours are [1,2,3,4,5] % values of 2D histograms. (**C**) Normalized distribution of the orientation of merging eddies; a bin reaching 0.2 means that it represents 20% of the total merging events for a given polarity. Errorbars show ± the standard deviation in each bin using a Monte Carlo Bootstrapping method with 10,000 re-sampling. (**D**) Same as (**C**) for the *Ro* ratio between merging eddies. (**E**) Same as (**C**) for the standard deviation of the Coriolis parameter outside eddies, divided by the mean value of Coriolis parameter in the same area —the ‘outside’ is defined as the area inside a circle of radius two times the distance between the merging eddies. (**F**) Same as (**C**) for the vorticity variance ratio between the areas outside and inside merging eddies, as defined in Eq. (4) —the ‘inside’ area corresponds to the area defined by the two eddy contours when the merging is detected. (**G**) Same as (**B**), for the number of neighbouring eddies detected outside eddies, depending on their polarity. Blue and red crosses indicate the mean number of neighbouring eddies for AEs and CEs respectively: on average, 0.93 (resp. 1.74) AEs, and 1.86 (resp. 0.92) CEs surround merging AEs (resp. CEs). (**H**) Position of the nearest edges of the neighbouring eddies; the grey scale indicates the value of a 2D histogram, and the black bold circle presents the chosen edge of the *β*-disk in idealized simulations. (**I**) Same as (**C**) for the merging distance *d*_*m*_ = *d*/*R*, where *d* is the Euclidean distance between the two eddy centers when they merge, and *R* is the average of the *R* values between the first detection of the eddy and the merger; dashed and dotted lines show the distributions of *d*_*m*_ for which *R* is equal to the mean value of *R* in each bin, plus or minus the standard deviation of *R* in this bin. Details about the calculation of quantities in each panel are presented in the ‘Methods’ section.

5,867 merging events were detected, *viz*. 605, 605, 1,797, 937, and 1923 in the NP, SP, NA, SA, and IO areas, respectively (Fig. 1). They were identified and extracted over a five year period, from the 1/12° HYCOM re-analysis dataset (hereafter ‘HYCOM dataset’, see ‘Methods’ section for the dataset, the detection algorithm, hereafter AMEDA, and Supplementary Fig. 2A and 9 for an example of merging event detection). The dataset relies on the assimilation of altimetric and *in situ* data in a mesoscale resolving numerical model, ensuring realistic surface fields with a satisfying horizontal resolution. 1,031, 1,037, 2,647, 1,445, and 2,779 different eddies were involved in the detected merging events, out of 4,051, 4,641, 6,079, 4,222, and 6,131 detected eddies (*i.e*. individual trajectories), thus implying a probability of an eddy undergoing a merging event of 25%, 22%, 44%, 34%, and 45% in the NP, SP, NA, SA, and IO areas, respectively. Eddies are thus more prone to merge in the NA and IO areas, because of the proximity of strong currents that generate numerous eddies^30^, *i.e*. the Gulf Stream and the Antarctic Circumpolar Current. Merging eddies have Rossby and Burger numbers (*Ro* and *Bu*, see ‘Methods’ section) in the ranges 0.03 < *Ro* < 0.1 and 0.1 < *Bu* < 1.5, with a probability density maximum at *Ro* = 0.05 and *Bu* = 0.5 (Fig. 2B). As *Ro* ≪ 1, estimations of the velocities from the geostrophic balance using the Sea Surface Height is accurate^36^. The upper limit of *Bu* corresponds to the limit of accurate eddy detection by AMEDA^37^. Eddies are not frontal (*Ro*∕*Bu* ≪ 1), meaning that there is no strong polarity bias in the stability of eddies^38^. Still, more mergers of anticyclonic eddies (AEs) than cyclonic eddies (CEs) are detected (Fig. 2A), independently of the number of detected eddies. However, results depend on the area considered, with maximum values of 56.4% and 54.6% for AEs merger respectively in the SP and in the SA (Supplementary Figs. 3–7). 60.4% and 54.9% of mergers involve AEs with *Bu* < 0.5 and 0.5 < *Bu* < 1 respectively, while the trend is reversed for *Bu* > 1, with 57.3% of mergers for CEs (Supplementary Fig. 8), suggesting that AEs with small *Bu* are more prone to merging than CEs. Because most of the merging events we detected involved eddies with *Bu* < 1, the global statistics reflect the behavior of eddies at small *Bu*. The orientation of the eddy pairs shows that a larger proportion of them are oriented meridionally (Fig. 2C). A CEs/AEs asymmetry is seen in the orientation, with more AEs (resp. CEs) with an orientation near 60° (resp. 120°). This indicates that when they merge, AE (resp. CE) pairs are more likely to be orientated Southwest-Northeast (resp. Northwest-Southeast). Note that the standard deviation is large because of the orientation variability observed between the different regions. This pattern has a clearer signature in the southern hemisphere than in the northern hemisphere, in particular in the SA and the IO areas (see Supplementary Figs. 6, 7), where AEs (resp. CEs) propagate northwestward (resp. southwestward)^39^. However, it would be speculative to attribute this CEs/AEs asymmetry to the *β*-drift propagation of eddies only, as other processes such as the eddy generation may play a role in the orientation of merging eddies.

These results provide important information to test the validity of usual assumptions used in isolated vortex merger studies. First, the ratio of the *Ro* of the two merging eddies (Fig. 2D), a proxy of the symmetry of the system, shows that a significant number (>10%) of merging events occur with one eddy having a Rossby number 1.5 times larger than the other eddy. Most of the merging events studied here are nonetheless symmetric, as about 45% of merging events have a *Ro* ratio lower than 1.2. This may also be related to the resolution of the HYCOM dataset, which does not allow for extreme asymmetric events (*e.g*. with *Ro* ratio > 2). Second, the Coriolis parameter varies by about 3–5% (Fig. 2E) around eddies: meridional asymmetry is intrinsic to the system. Third, the vorticity variance (Fig. 2F) around eddies is important for a significant number of merging events. For isolated merging events, the latter is zero, because no background flow is present. Thus, merging eddies are not isolated, and the merging process is influenced by other structures located close to the two merging eddies. In particular, other coherent eddies are detected in the periphery of merging eddies (Fig. 2G), with an average of two CEs (resp. AEs) detected around merging AEs (resp. CEs). The polarity asymmetry in the number of neighbouring eddies results from the CE/AE asymmetry due to previous merging events (*i.e*. close eddies with the same polarity are more likely to have already merged). Surrounding eddies are close to the merging eddies: their median edge is located at 4.5*R* from the centroid of the interacting pair (Fig. 2H). Merging eddies are thus *constrained* inside a sea of eddies. Note here that AMEDA does not take into account 3-eddy merging events. It thus underestimates the number of merging events, and maybe overestimates the number of like-signed eddies around the two merging eddies. In summary, the usual hypotheses for symmetric, isolated vortex merger —*i.e*. two identical eddies on the *f*-plane, isolated from the surrounding— are not verified in the HYCOM dataset.

### Comparing the merging distance between the global ocean and idealized simulations

**Figure 3:**
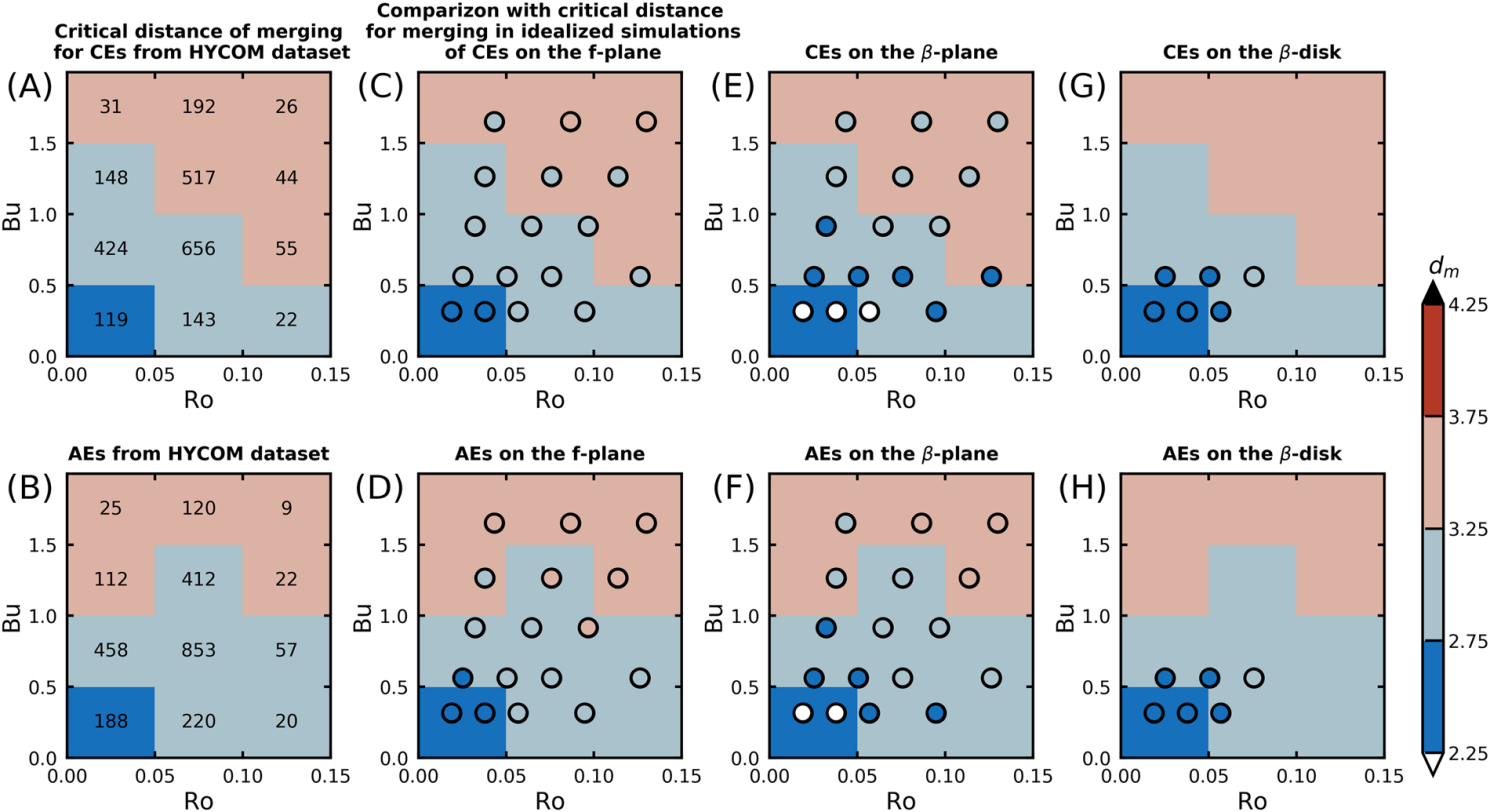
Comparison between the mean distance of merging in the HYCOM dataset, and the maximal initial distance between eddies for which they merge in the idealized simulations. (**A**) Mean distance of merging *d*_*m*_ for CEs in the HYCOM dataset, depending on *Ro* and *Bu*. The numbers of values used to compute averages are indicated for each range of *Ro* and *Bu*. (**B**) Same as (**A**) for AEs in the HYCOM dataset. (**C**) Maximum distance of merging ${d}_{m}^{i}$ observed in numerical simulations of CEs with *α* = 2 and *H* = 1000 m on the *f*-plane (the results are shown by the circle colors). The color background is the same as in (**A**) to allow a comparison between numerical simulations and the HYCOM dataset analysis. (**D**) Same as (**C**) for AEs on the *f*-plane. (**E**) Same as (**C**) for CEs on the *β*-plane. (**F**) Same as (**C**) for AEs on the *β*-plane. (**G**) Same as (**C**) for CEs on the *β*-disk. (**H**) Same as (**C**) for AEs on the *β*-disk.

In the HYCOM dataset, the average normalized merging distance *d*_*m*_ is of 3.04 for AEs and 3.09 for CEs (Fig. 2I). This distance is obtained on the first day of interaction between the merging eddies, when they start to influence each other (see ‘Methods’ section). It thus reflects the critical distance below which their velocity fields interfere constructively, ultimately causing the collapse of the vortex system. This distance depends on the *Ro* and *Bu* of merging eddies (Fig. 3A,B). *d*_*m*_ increases with *Ro* and *Bu*, such that *d*_*m*_ ≈ 2.5 for *Ro* < 0.05 and *Bu* < 0.5, and *d*_*m*_ ≈ 3.5 for *Ro* > 0.1 and *Bu* > 1. For a given eddy radius (*i.e* a constant *Bu*), *Ro* increases with the maximal swirl velocity of the eddy. As the latter becomes larger, the advective timescale induced by the eddies decreases, causing a quicker collapse of the eddies. This leads to increasing the minimum distance for which eddies can interact and merge, and explains the increase of *d*_*m*_ with *Ro*. The variations of *d*_*m*_ as a function of *Bu* are related to the baroclinic component of the flow.

In idealized simulations, merger strongly depends on the *Ro* and the *Bu* of eddies at initialization (Figs. 3C–H, see ‘Methods’ section for details about simulations, and Supplementary Figs. 2B,C and 10,11 for example of merging events in these simulations). We compute the maximum initial distance between the two eddies at which merging occurs ${d}_{m}^{i}$. The latter increases with *Ro* and *Bu*, and merging is thus easier for smaller eddies than for large ones. Independently of other parameters, the radial steepness of the velocity profile *α* and the height of the eddies *H* have a strong influence on the interaction between eddies (Supplementary Fig.12). Indeed, the steeper the eddy velocity profile is (or/and thinner it is), the smaller ${d}_{m}^{i}$ is: (1) eddies with smaller *α* interact with farther structures; (2) as *H* decreases, the baroclinic modes take over the barotropic mode, therefore decreasing its horizontal influence^21,22^. On the *f*-plane, a clear AEs/CEs asymmetry is evident: ${d}_{m}^{i}$ is larger for AEs than for CEs. Using the cyclogeostrophic balance, for a given velocity field, a given pressure anomaly has a larger extent for AEs than for CEs, causing AEs to interact at larger distances.

The *β*-effect reduces ${d}_{m}^{i}$, especially for larger eddies. The physical mechanism responsible for the decrease of ${d}_{m}^{i}$ can be described as follows. With the *β*-effect, meridionally aligned eddies generate a Rossby wave composed of meridionally elongated Sea Surface Height patches with zonally alternating sign (*e.g* Supplementary Fig. 2C). This wave creates a southward (resp. northward) velocity east of the two AEs (resp. CEs); thus, the southern (resp. northern) eddy moves southward (resp. northward), weakening the merging efficiency. Supplementary Figs. 10,11 illustrate this phenomenon. The opposite situation would happen if one has considered a situation in the southern hemisphere. Modifying the orientation of the two eddies at initialization (not shown) tends to slightly reduce this effect. However, even when eddies are zonally aligned, an intense shear generated by Rossby waves leads to the stretching of the two eddies along the zonal axis. This also acts in reducing the merging efficiency.

Running simulations with *β*-effect and a circular wall around the eddies (*β*-disk simulations, see ‘Methods’ section), allows to simulate the situation where solitary eddies drift during a long time period^39^, and are then constrained by neighbouring eddies when they interact —the free-slip wall acts as an image vortex^40^, and prevent the eddies to drift away. In such simulations, merging eddies can exchange more vorticity as the wall prevents them to drift far away. This increases the merging efficiency as well as ${d}_{m}^{i}$.

The merging distances observed in the HYCOM dataset are reproduced by the idealized simulations on the *f*-plane: the values of ${d}_{m}^{i}$ match well the observed *d*_*m*_ for all *Ro* and *Bu* studied (in particular for simulations with *H* = 1000 m and *α* = 2, as expected —in the ocean, eddies are mostly Gaussian^37^, and about 1000 m deep^41–44^). With the *β*-effect, if eddies are not constrained by a disk, *d*_*m*_ in the HYCOM dataset is significantly larger than the ${d}_{m}^{i}$ values observed in idealized simulations, especially for small *Bu* and *Ro*. In this parameter range, *β*-disk simulations with *H* = 1000 m and *α* = 2 are found closer to the measures made in the HYCOM dataset.

## Discussion

The hypotheses of ‘isolated vortex merger’ studies are rarely verified in the global ocean. First, a significant number of merging situations are not symmetric. Some merging events may thus be influenced by ’vortex thinning’ events^45^, where the largest eddy causes the smallest one to wrap around it^46^. This supports the view of ref. ^47^, that vortex thinning rather than vortex merger may play a significant role in the inverse energy cascade. Second, the *β*-effect plays an important role in the merging process. It modifies the shapes and motions of eddies before the merger, as they drift westward instead of co-rotating. Third, opposite-signed coherent structures were found close to most merging eddies. This result together with the findings of ref. ^48^, which showed that isolated eddies are surrounded by opposite-signed coherent structures, shows that the turbulent open ocean is thus closer to a dense ‘sea of modons’^49^ rather than pairs of isolated eddies. This is a key result since opposite-sign eddies can couple with any of the merging eddies, thus reducing or increasing their critical distance of interaction, and modifying momentum transfer between them^28^. After merger, eddies are stabilized by such dipolar couplings. Also, as eddies are surrounded by neighbouring eddies, they can not drift as freely as if they were isolated: the merger is constrained in a finite area. The physical properties of the flow during the merger significantly differ from those observed in isolated vortex merger studies.

Comparison between the critical distance observed in the global ocean and the values found in idealized simulations highlights a paradox with strong implication on the understanding of vortex merger in the global ocean. The critical distances of merging found for *f*-plane simulations involving 1000 m deep Gaussian eddies are close to the observed distance of merging in the global ocean (for the present range of *Ro* and *Bu*). Furthermore, the mean critical distance of 3.05*R* found in the global ocean is consistent with values found in the literature when considering 3D quasi-geostrophic simulations of two isolated vortices on an *f*-plane^21,22^. As merger is influenced by the *β*-effect, one would expect that considering *β*-plane simulations would give a better accuracy in the merger distance estimation. We observe the opposite, with a distance of merger significantly lower in *β*-plane simulations than observed in the ocean. Other mechanisms are thus at work, which tend to increase the critical distance of merging. The results of our simulations suggest that these mechanisms are related to the proximity of neighbouring eddies, which prevent the eddies to drift away —by *β*-effect— during the merger. Indeed, if we consider that the merger is constrained in a finite area, on the *β*-plane, the critical distance is found to be closer to the global ocean analysis. Furthermore, in the real ocean, merger may also be impacted by the coupling of one or two of the eddies with opposite-signed companions. Considering all the actual parameters in the ocean, the vortex merger is thus best described by *non*-isolated idealized situations. This paradox shows that focusing on the critical distance of merging to describe merging events in the ocean may lead to a misevaluation of the physical mechanisms involved in the merger.

The numerous merger we studied, together with previous studies^6–11^, suggests that the merger of large mesoscale eddies are key for the transfer of heat and energy to large scales, through the mixing of water masses in surface and sub-surface. Recent studies suggested that, in the ocean, the number of vortex splitting is comparable to that of the vortex merger^30^. This other kind of events should therefore play an important role in the ocean energy budget as well, opposite to the role of the vortex merger, as it transfers heat and energy toward small scales. A similar study as the one presented here could thus be conducted, in order to describe the modalities of the splitting process in the global ocean. In particular, the AMEDA algorithm used here is suitable for such a task, as it allows to get the historic of splitting events that eddies went through, exactly similarly as for the merger^37^.

To accurately describe the vortex splitting, as well as to extend the present study to smaller vortices (*i.e*. for larger *Ro* and *Bu*), a higher-resolution dataset should be used. Indeed, with a higher resolution, smaller vortices are detected. The study of smaller structures could allow to describe the interaction regimes between eddies at horizontal scales below the mesoscale. Using a regional model in several regions of the ocean, rather than a global one, could ensure to have a correct representation of both the mesoscale and the submesoscale dynamics, and therefore an unprecedent description of the vortex-vortex interactions.

## Methods

### Global ocean analysis, and the definition of the five study areas

We use the Sea Surface Height and the surface horizontal velocities in the period 01/01/2000 to 31/12/2004, in the five study areas shown in Fig. 1, from the GOFS 3.1, 41-layer HYCOM+NCODA Global 1/12° re-analysis. The product is supplied every three hours on a 0.08° resolution Mercator grid between 40°S and 40°N. For our study, a five-year period is sufficient to obtain robust statistics. We consider one output per day for our study, to decrease the computational time.

This HYCOM re-analysis data have been intensively validated^50^ and used^51–54^ in several contexts during the past ten years. The global HYCOM re-analysis uses the Navy Coupled Ocean Data Assimilation (NCODA) system^55,56^ for data assimilation. NCODA uses the 24-hour HYCOM forecast^57^ as a first guess in a 3D variational scheme and assimilates available satellite altimeter observations, satellite, and *in situ* sea surface temperature as well as *in situ* vertical temperature and salinity profiles from XBTs, Argo floats and moored buoys. Surface information is projected downward into the water column using Improved Synthetic Ocean Profiles^58^.

We focus on areas respecting the hypothesis of isolated vortex merger studies. First, areas are far away from the coastlines, topographic features, and strong currents (to avoid coastal, topographic, or current/eddy interactions). We also exclude areas close to islands —this explains blank areas *e.g*. in the northern part of the SP area around Marquesas Islands. Second, the five areas are located at mid-latitudes (to avoid equatorial dynamics and ice related phenomena). The mean values of the Coriolis frequency *f*, of the depth of the seafloor, and of the first baroclinic Rossby radius of deformation *R*_*D*_ ~ 45 km^59^ are similar in the five areas. Also, as revealed by Argo floats^60^, the mean annual stratification (represented by the Brunt-Väisälä frequency) of the five areas are similar (Supplementary Fig. 1). The characteristics of merging events vary little between the study areas (Supplementary Figs. 3–7). We thus mostly consider statistics over all areas. Note that the five study areas are similar to the one discussed in ref. ^48^.

### Numerical simulations of vortex merger

#### Numerical setup

We perform idealized simulations of isolated vortex merger using the Coastal and Regional Ocean COmmunity model (CROCO)^61^. This model solves the hydrostatic primitive equations for the velocity, temperature, and salinity, using a full equation of state for seawater^62^. The simulations performed integrate the primitive equations for 50 days. The time step is 900 s. The horizontal domain size is 1200 × 1200 km. The bottom is flat, at 3000 m depth. The boundaries of the domain do not affect the dynamics of the eddies that are surface intensified and initially set at the center of the domain. The horizontal resolution is 5 km. 32 vertical levels are chosen, stretched at the surface. The coarse resolution is consciously chosen since our aim is to perform numerous simulations to cover up as well as possible the parameters space. Simulations performed on the *f*-plane use Coriolis parameter *f*_0_ = 6.6 × 10^−5^ s^−1^; simulations on the *β*-plane use a linearly varying Coriolis parameter *f* = *f*_0_ + *βy*, with *y* the meridional coordinate and *β* = 2 × 10^−11^ m^−1^ s^−1^. *f*_0_ and *β* are the mean value of the 5 study areas, and are representative of the northern hemisphere mid-latitude dynamics.

The numerical settings are similar to previous simulations performed in an idealized context^63,64^: horizontal advection terms for tracers and momentum are discretized with fifth-order upwind advection schemes (UP5); the explicit horizontal viscosity and diffusivity are set to zero, since the UP5 scheme damps dispersive errors; the vertical advection is discretized with a fourth-order centered parabolic spline reconstruction (Splines scheme). Further discussion about these parameterizations can be found in^63^ or^65^. No vertical diffusion is added; this allows to avoid numerically parameterized diabatic effects, which can affect the conservation of Potential Vorticity^66^.

#### Simulations initialization

We initialize an analytical background stratification *N*(*z*), which fits the average ambient stratification in the five selected study areas (Supplementary Fig. 1):

1 $$N(z)={N}_{0}+{N}_{1}\ {e}^{z/{z}_{h}},$$

with *z* < 0 the vertical coordinate, *N*_1_ = 9 × 10^−3^ s^−1^, *z*_*h*_ = 150 m, and *N*_0_ = 7 × 10^−3^ s^−1^. Integrating this stratification from the surface (where *ρ*(*z* = 0) = 1030 kg m^−3^), gives the ambient density background *ρ*(*z*); the temperature background *T*(*z*) is obtained by inverting the TEOS-10 equation of state for seawater^67^ and assuming a constant salinity background *S*(*z*) = 35 psu. The model is initialized with these temperature and salinity background profiles.

We initialize surface intensified eddies of given relative vorticity profile. For each eddy, we set the initial profile of surface vorticity:

2 $$\omega (r)=\pm {\omega }_{0}\ {e}^{-{\left(\frac{r}{R}\right)}^{\alpha }},$$

with the sign depending on the eddy polarity, $r=\sqrt{{(x-{x}_{0})}^{2}+{(y-{y}_{0})}^{2}}$ the radial coordinate referenced at the center of the eddy (*x*_0_, *y*_0_), and *α* the steepness parameter. *ω*_0_ = *V*/*R*, with *V* the maximal azimuthal velocity at a distance *R* from the center of the eddy. The surface azimuthal velocity of the eddy is computed using ${v}_{\theta }(r,0)=\frac{1}{r}\int dr\ \omega \ r$. By definition, this velocity vanishes slowly with *r*, contrary to *e.g*. Rankine vortices. To avoid the presence of spurious velocity at the edges of the domain, we apply a Hanning window on *v*_*θ*_ to make it smoothly tend to zero at *r* > 3 *R*. The horizontal velocity decreases at depth such that *v*_*θ*_(*x*, *y*, *z*) = *v*_*θ*_(*x*, *y*, 0) *e*^−*z* ∕*H*^, thus defining the height of the eddy *H*. The horizontal velocity field of the eddy (*u*, *v*) is obtained by projecting *v*_*θ*_ into Cartesian coordinates. The pressure anomaly field ${P}^{{\prime} }(x,y,z)$ corresponding to this velocity field is computed *via* the Gradient Wind equation:

3 $$2J(u,v)+f({\partial }_{x}v-{\partial }_{y}u)=\frac{1}{\rho }{\Delta }_{h}{P}^{{\prime} },$$

with *J*(*u*, *v*) = ∂_*x*_*u*∂_*y*_*v* − ∂_*y*_*u*∂_*x*_*v* the Jacobian operator, and Δ_*h*_ the horizontal Laplacian operator. From ${P}^{{\prime} }$ we determine the density and the temperature anomalies of the eddy. These anomalies are computed for the two eddies, at positions *x*_0_ = *x*_*c*_, and *y*_0_ = *y*_*c*_ ± *d*/2, thus defining the initial distance between the eddies *d*. *x*_*c*_ and *y*_*c*_ are the coordinates of the center of the domain. Note that the eddies are stable during the whole simulation.

This initialization uses only few parameters for eddies: *α* defines the steepness of the velocity profile (*α* = 2 corresponding to a Gaussian vortex); *H* is related to the baroclinicity of the eddy. For our study, we vary the initial parameters as *R* = [35, 40, 47, 60, 80] km, *V* = [0.1, 0.2, 0.3, 0.5, 0.7]m s^−1^, *α* = [2, 4], and *H* = [500, 1000] m, for both cyclonic and anticyclonic eddies. For each set of parameters, we also vary the initial distance between the two eddies in the range *d*/*R* = [2.5, 3.0, 3.5, 4.0], and we run simulations on both the *f*-plane and the *β*-plane. Hence, we are able to know the maximum value of *d*/*R* (with an accuracy of ±0.25) for which eddies with given initial parameters merge (this value is noted ${d}_{m}^{i}$ in the text). We scan 400 different sets of parameters, and a total of 1600 simulations. We downgrade the resolution of the outputs to 0.08°, and we consider one output per day to have a dataset consistent with the HYCOM outputs.

To study the vortex merger in a context where eddies are not isolated but packed, surrounded by neighbouring eddies, we perform a few simulations with a boundary around the two main eddies. The boundary is a circular wall with a radius of 4.5*R* from the center of the domain. These simulations are referred to as *β*-disk simulations in the text. The radius of the disk is chosen as the median distance between the merging eddies centroid and the closest neighbouring eddies edge (see Fig. 2H). A free-slip condition is chosen such as no frictional effect are induced by the boundary.

### Our definition of vortex merging events with AMEDA

To identify the merging events both in the HYCOM dataset and in idealized simulations, the Angular Momentum Eddy Detection and tracking Algorithm (AMEDA^37^) is used. One of the benefits of AMEDA is that it does not depend on arbitrary thresholding, which would require a fine-tuning of geometrical parameters. Also, the algorithm is robust with respect to the grid resolution and can thus be applied to a wide variety of velocity fields (experimental, numerical, altimetry...). This algorithm has been used and validated in previous studies in the past few years^36,37,44,68^, see also an example of application of AMEDA in https://www1.lmd.polytechnique.fr/dyned/. To detect eddies, we use the Sea Surface Height, to avoid false detections due to mixed-layer related phenomena. Note that considering only the Sea Surface Height may underestimate the velocity of detected eddies since the algorithm only takes into account the geostrophic part of the velocity field. A full description of the algorithm is presented in Fig. 1 and Fig. 13 of^37^. In particular, the detection of merging works as follows: the algorithm identifies the couples of eddy centers that are encompassed by a closed streamline (*i.e* a shared contour), and it checks if one of the two eddy trajectories ends after the interaction period. If these two conditions are met, the merging event is counted and one can access the characteristics of the two eddies when merging occurs.

This method provides the characteristics of each detected eddy from its generation (or first detection) to the last time it was detected (either its death due to destabilization or interaction with broader scale structures (topography, currents...), or until it merges with another eddy): the position of the eddy center, its shape, its polarity, its radius *R* (defined as the mean radius of the maximum velocity contour), the mean maximum velocity along this contour *V*, and the ID of eddies that would have merged with it. Example of merging events are shown for the HYCOM dataset, an *f*-plane simulation, and a *β*-plane simulation in Supplementary Figs. 2A–C respectively.

The definition of merger is the same for both the HYCOM dataset and the idealized simulations. As discussed in previous studies^21,25^, defining whether merging occurred may be tricky, mainly for three reasons. First, they can rotate around each other and exchange momentum, but keep two vorticity maxima. The distinction between close co-rotating eddies and a single eddy resulting from the merger can thus be arduous. Second, in the case of Gaussian eddies, two co-rotating eddies will always merge after an infinite period of time. Third, several types of regimes can be observed when co-rotating vortex pairs interact: elastic interaction, complete merger, partial merger, partial straining out, and complete straining out^31,46,69,70^. For this study, we abstain from these ambiguities by considering that merging occurred if AMEDA detected a merging event during the lifetime of detected eddies. We thus only differentiate a merging event *versus* no merging event, similarly as ref. ^23^. In the case of partial merger, the detection of the merger will depend on the merger efficiency. Indeed, as this one increases, the smallest eddy is too small to be detected, and AMEDA indicates a merging event. We do this in both HYCOM dataset and in idealized simulations, thus giving a consistent definition of merging in the two cases. In the case of the idealized simulations, if eddies have not merged during the first 50 days of simulation, they are considered as independent. This duration has been chosen knowing that the median lifetime of eddies before merging in the HYCOM dataset is 40 days. Note that in the HYCOM dataset, to be sure that we consider merging events of two stable vortices, we only consider eddies with a lifetime larger than 10 days.

### Common definitions: dimensionless numbers and distance of merging

The definitions of *R* and *V* in both the initialization of idealized simulations, and AMEDA detections in the HYCOM dataset are consistent. From *R* and *V*, one can compute the Rossby and Burger numbers of the merging eddies. They are respectively defined as *Ro* = *V*/(*f*_0_ *R*) and $Bu={({R}_{D}/R)}^{2}$. *Ro* is a proxy of the surface intensity of the dynamic core^1^; *Bu* indicates the importance of stratification against rotation. The values of *R* and *V* chosen for the initialization of the idealized simulations give a range of Rossby numbers varying between *Ro* = 0.019 and *Ro* = 0.3, and Burger numbers between *Bu* = 0.32 and *Bu* = 1.65. This covers the parameter space of eddies which merge in the HYCOM dataset (see Fig. 2B). Note that for the study of eddies detected in the HYCOM dataset, *R* is in fact the average of the *R* values between the first detection of the eddy and the merger. This average is done because the radius of eddies before the merging may vary substantially due to the strong interactions between eddies (see *e.g*. Supplementary Fig. 2). To compute *Bu* for each detected eddy in the HYCOM dataset, we interpolate the value of *R*_*D*_ from the ref. ^59^’s global estimation at the position of the detected eddy.

The normalized distance between eddies when they merge in the HYCOM dataset *d*_*m*_ is defined as the Euclidean distance between the two eddy centers (Fig. 2I) when AMEDA detects the merger (on the first day of interaction between the two merging eddies). It is normalized by the mean radius of the two eddies. Since the two radii may differ, and because their value is impacted by the merging events, *d*_*m*_ is subject to variations. Thus, we present also in Fig. 2I the distributions of *d*_*m*_, for which *R* is equal to the mean value of *R* in each bin, plus or minus the standard deviation of *R* in this bin. They are respectively the dotted and the dashed step lines. *d*_*m*_ is defined such as it can be compared with the maximum initial distance between eddies, for which they are observed to merge in idealized simulations ${d}_{m}^{i}$ (for a given set of parameters).

### Other quantities used to describe merging events in the HYCOM dataset

From the eddy characteristics extracted by AMEDA in the HYCOM dataset, we compute quantities to describe the merging events occurring in the global ocean (Fig. 2). In the following we denote by •_1_ and •_2_ the quantities corresponding to the two eddies implied in a specific merging event.

We compute the ratio of the Rossby numbers for each pair of eddies (Fig. 2D) as $\max (R{o}_{1},R{o}_{2})$/$\min (R{o}_{1},R{o}_{2})$. Thus, for each pair, this ratio is always greater than 1, with 1 corresponding to eddies with equal Rossby numbers.

The orientation of the two eddies with respect to the meridional direction (Fig. 2) is computed from the positions of the two eddies on a Mercator projection. The orientation is computed modulo 180° so that the angle does not depend on which eddy is east or west of the system.

The other quantities described hereafter are computed in two particular domains around eddies. These two domains are specified in the text and in Fig. 2F,G as ‘inside’ and ‘outside’. The ‘inside’ area corresponds to the area defined by the two eddy contours when the merging is detected, hereafter ${{\mathcal{C}}}_{1}$ and ${{\mathcal{C}}}_{2}$ (see red bold contours in Supplementary Fig. 2A). The ‘outside’ is defined as the area inside a circle of radius two times the distance between the merging eddies, hereafter ${\mathcal{D}}$ (see black thin line in Supplementary Fig. 2A), from which we subtract the inside area. With these definitions of areas we compute the ratio of vorticity variance (Fig. 2F) as:

4 $$\frac{{\int }_{{\mathcal{D}}}dx\ dy\ {\omega }_{s}^{2}(x,y)-{\int }_{{{\mathcal{C}}}_{1}+{{\mathcal{C}}}_{2}}dx\ dy\ {\omega }_{s}^{2}(x,y)}{{\int }_{{{\mathcal{C}}}_{1}+{{\mathcal{C}}}_{2}}dx\ dy\ {\omega }_{s}^{2}(x,y)},$$

with *ω*_*s*_(*x*, *y*) = ∂_*x*_*v* − ∂_*y*_*u* the relative vorticity at the surface. This quantity thus describes whether the environment of the two merging eddies is turbulent or not. To be more specific about this point, we also count the number of neighbouring eddies detected by AMEDA for which the center is located in the outside area (Fig. 2G). The distance between the centroid of the merging eddies and nearest edge of neighbouring eddies is computed (Fig. 2H). For each neighbouring eddy, it is defined as the distance between the centroid of the merging eddies and the center of the neighbour, from which we subtract the radius of the neighbour. As this distance depends on the radius of merging eddies, it is normalized by *R*.

Also, we compute the variation of the Coriolis frequency Δ*f*/*f* around eddies (Fig. 2E) by computing the standard deviation of *f*(*x*, *y*) inside the ${\mathcal{D}}$ contour, divided by the mean value of *f*(*x*, *y*) in the same area.

## Supplementary information

Supplementary Information.
