# Supplementary material for: Oceanic vortex mergers are not isolated but influenced by the β-effect and surrounding eddies
Source: Sci Rep. 2020 Feb 19;10:2897. doi: 10.1038/s41598-020-59800-y (PMC7031327; doi:10.1038/s41598-020-59800-y)
Supplement: Supplementary file 1 — Supplementary Information. [file 41598_2020_59800_MOESM1_ESM.pdf]

Supplementary information for "Oceanic vortex mergers are not isolated but influenced by the β-effect and surrounding eddies"

Charly de Marez, Xavier Carton, Pierre L'Hégaret, Thomas Meunier, Alexandre Stegner, Briac Le Vu, and Mathieu Morvan

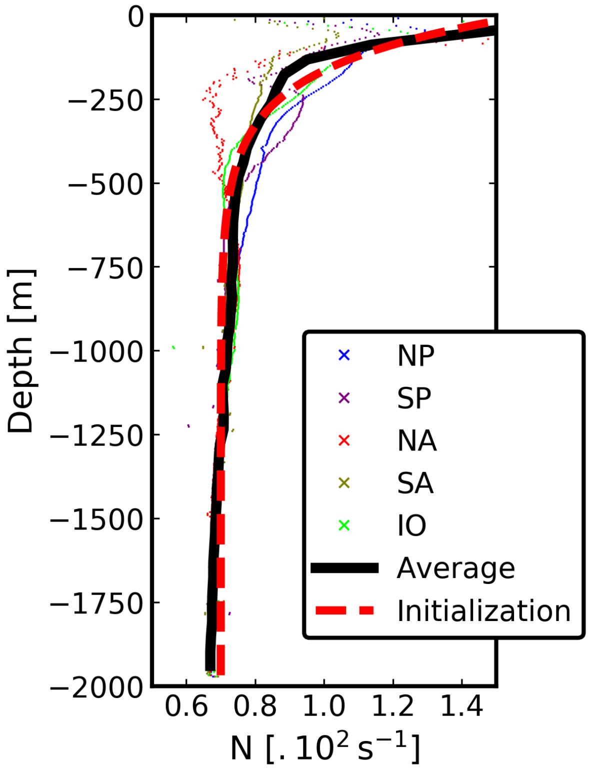

Figure 1: Brunt-Väisälä frequency N profiles in the five study areas. Black bold lines are the average profiles of each quantity over the five areas. Red dashed lines are the profiles used for the initialization of the idealized simulations. Profile in areas NP, SP, NA, SA, and IO are averaged over all available Argo profiles on the 2000-2005 period in each area. This represents respectively 11355, 11580, 6437, 4473, and 7084 profiles from 195, 221, 178, 97, and 165 different Argo floats.

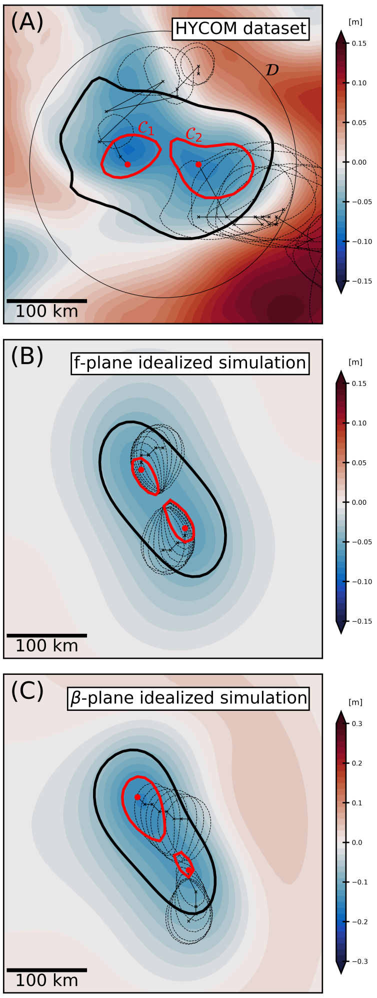

Figure 2: Example of merging detection with AMEDA, in the HYCOM dataset (A), in idealized simulations on the f-plane (B), and on the β-plane (C). The color background is the Sea Surface Height (at the merging moment) used by AMEDA for the eddy detection and tracking. Black crosses and black dashed lines are centers and contours of the two eddies before the merging event; red dots and red bold lines are the centers and the contours of the two eddies when merging occurs (as defined by AMEDA); the black bold contour is the contour of the eddy resulting of the merging (here 2 days after the merging event). Note that the domain shown in B and C is not the whole simulation domain (which is three times larger in both directions).

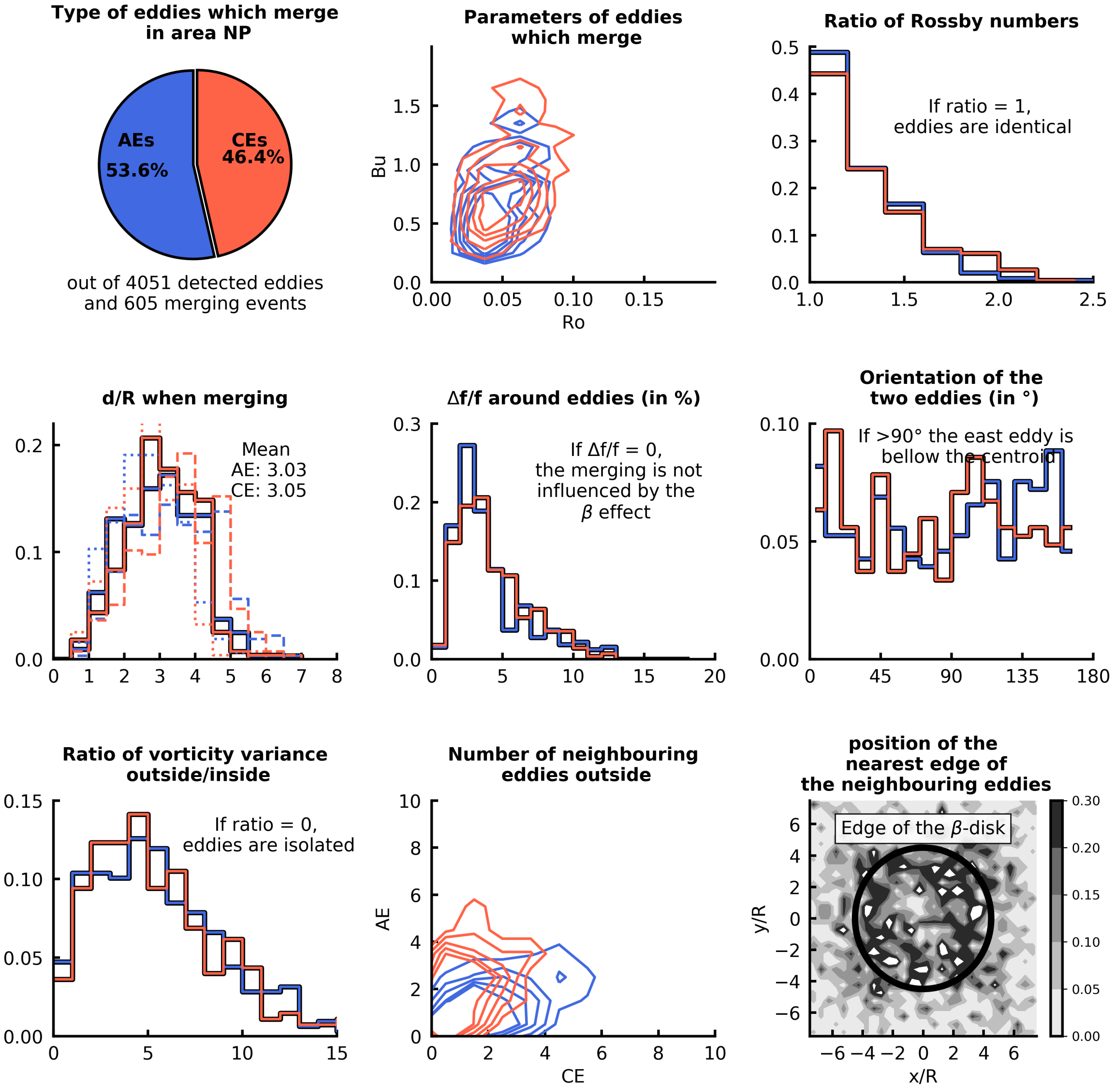

Figure 3: Same as Fig. 2 of the main manuscript, but for area NP

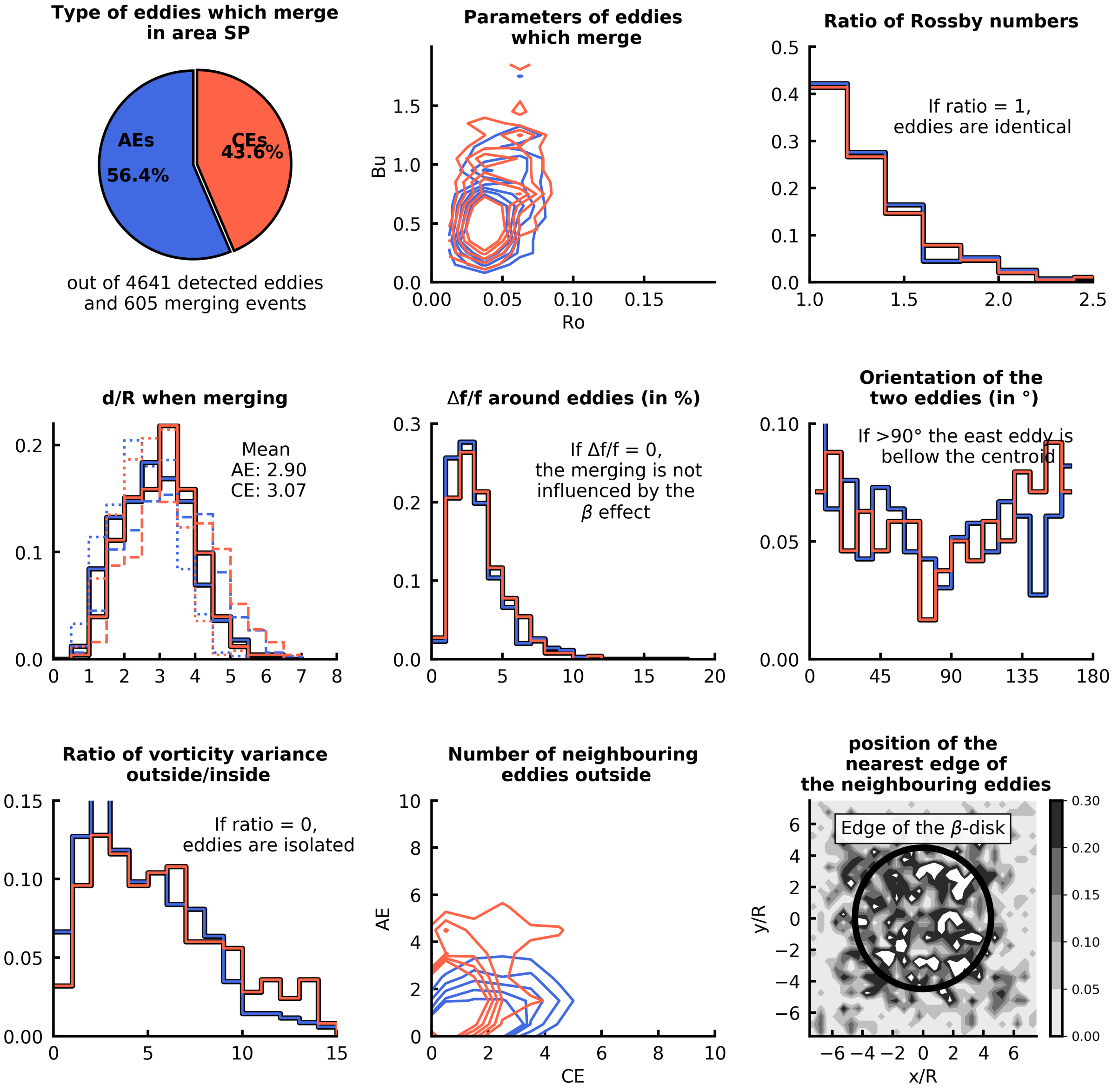

Figure 4: Same as Fig. 2 of the main manuscript, but for area SP

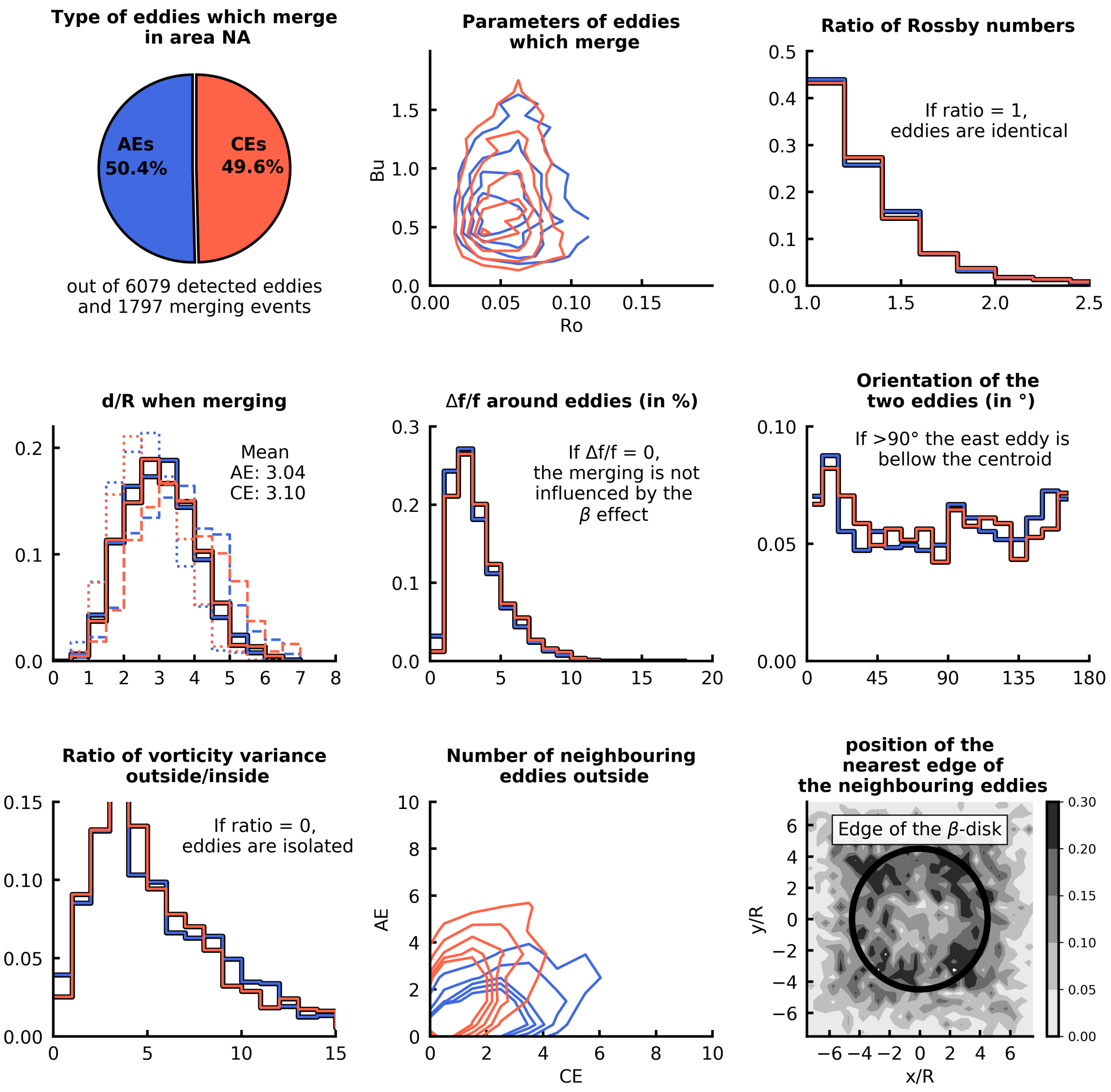

Figure 5: Same as Fig. 2 of the main manuscript, but for area NA

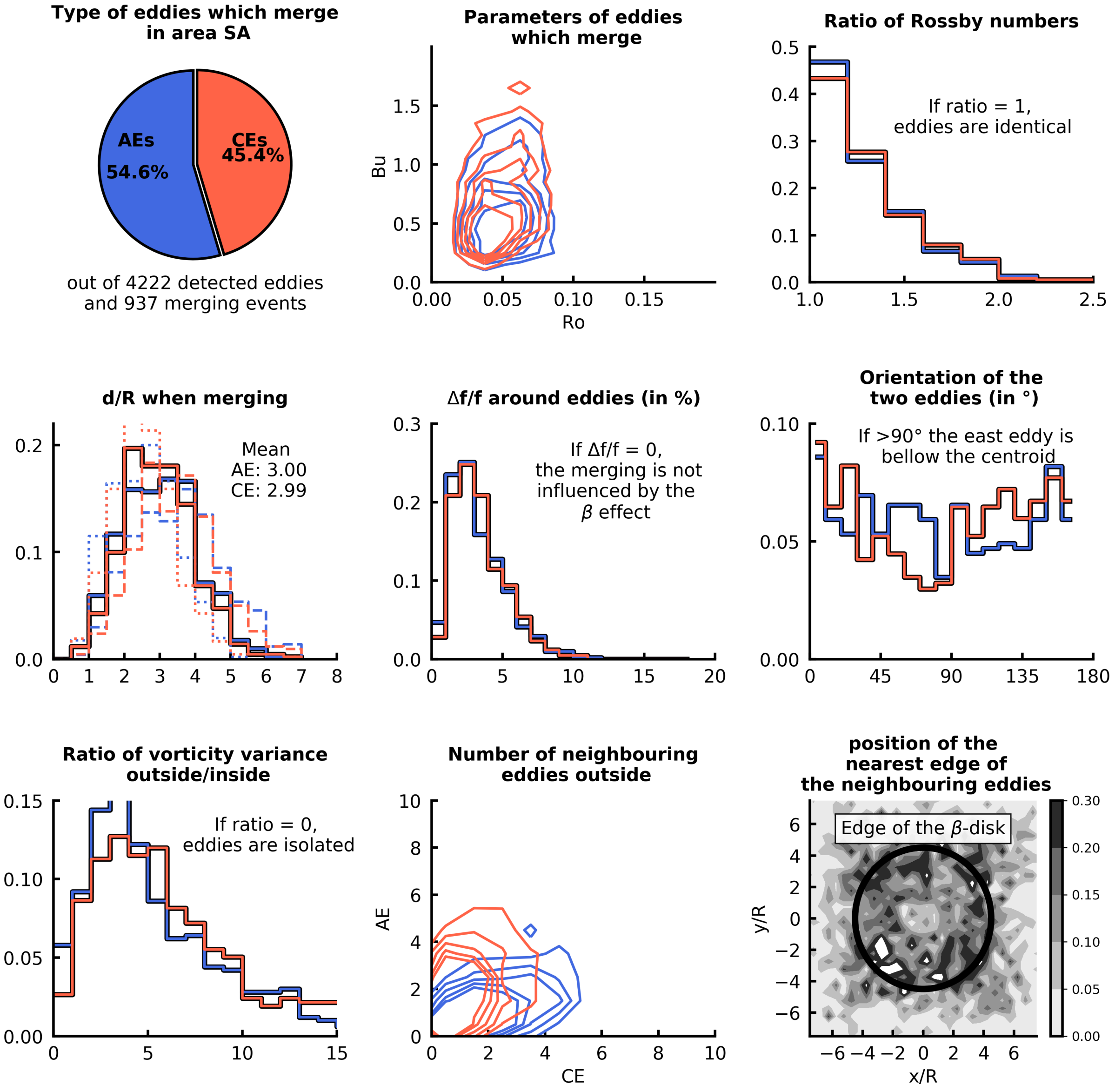

Figure 6: Same as Fig. 2 of the main manuscript, but for area SA

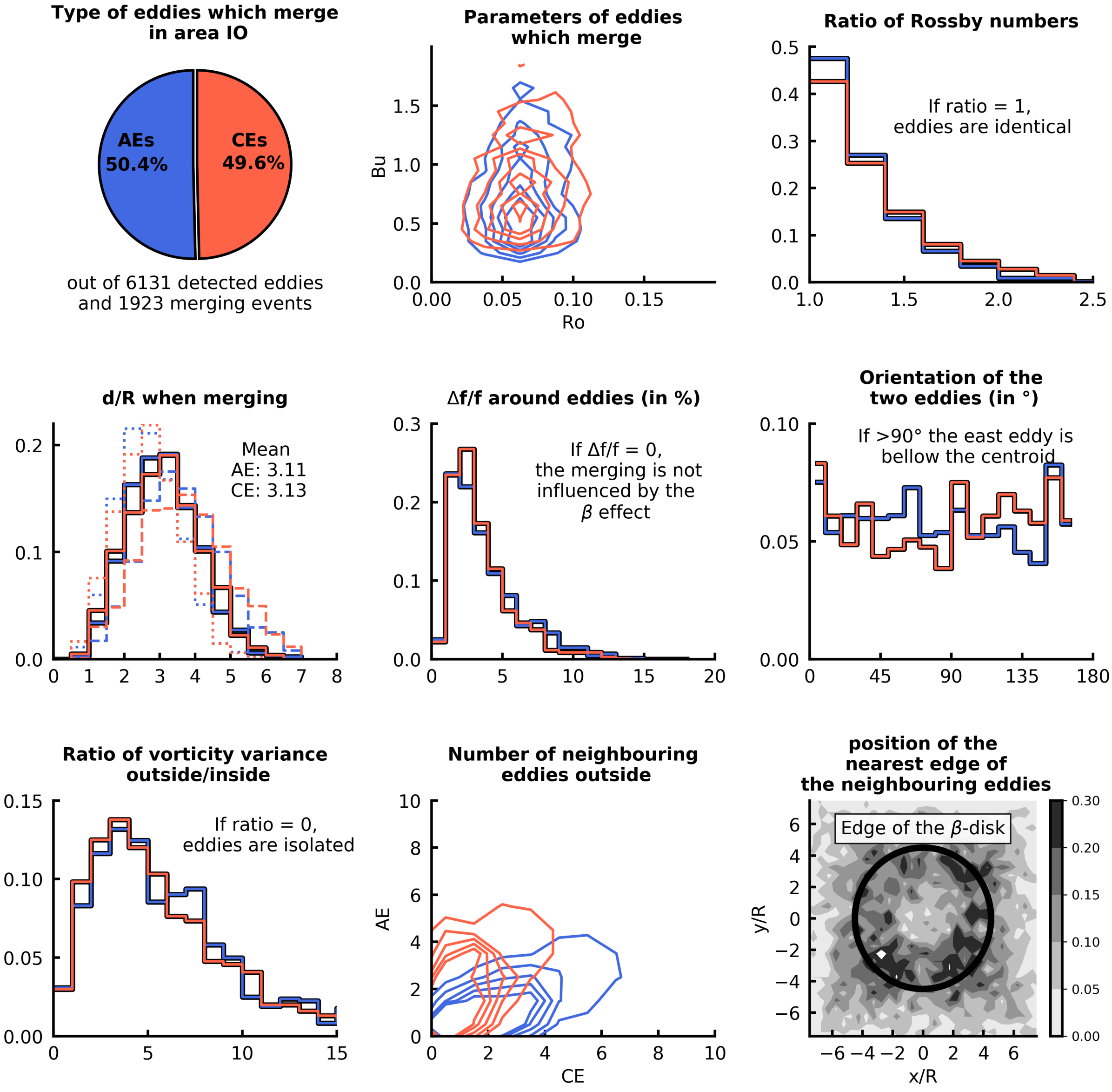

Figure 7: Same as Fig. 2 of the main manuscript, but for area IO

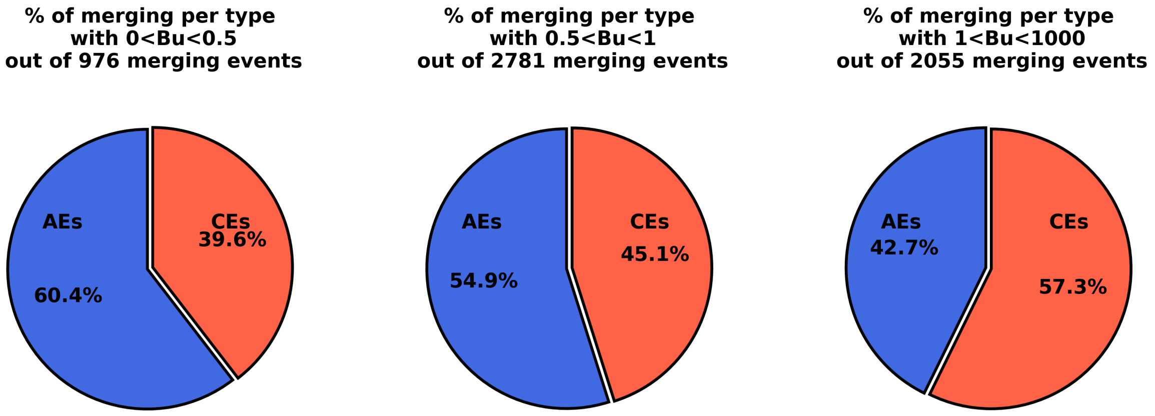

Figure 8: Same as Fig. 2A of the main manuscript, but only for merging eddies with $0 < Bu < 0.5$ (left), $0.5 < Bu < 1$ (middle), $1 < Bu$ (right).

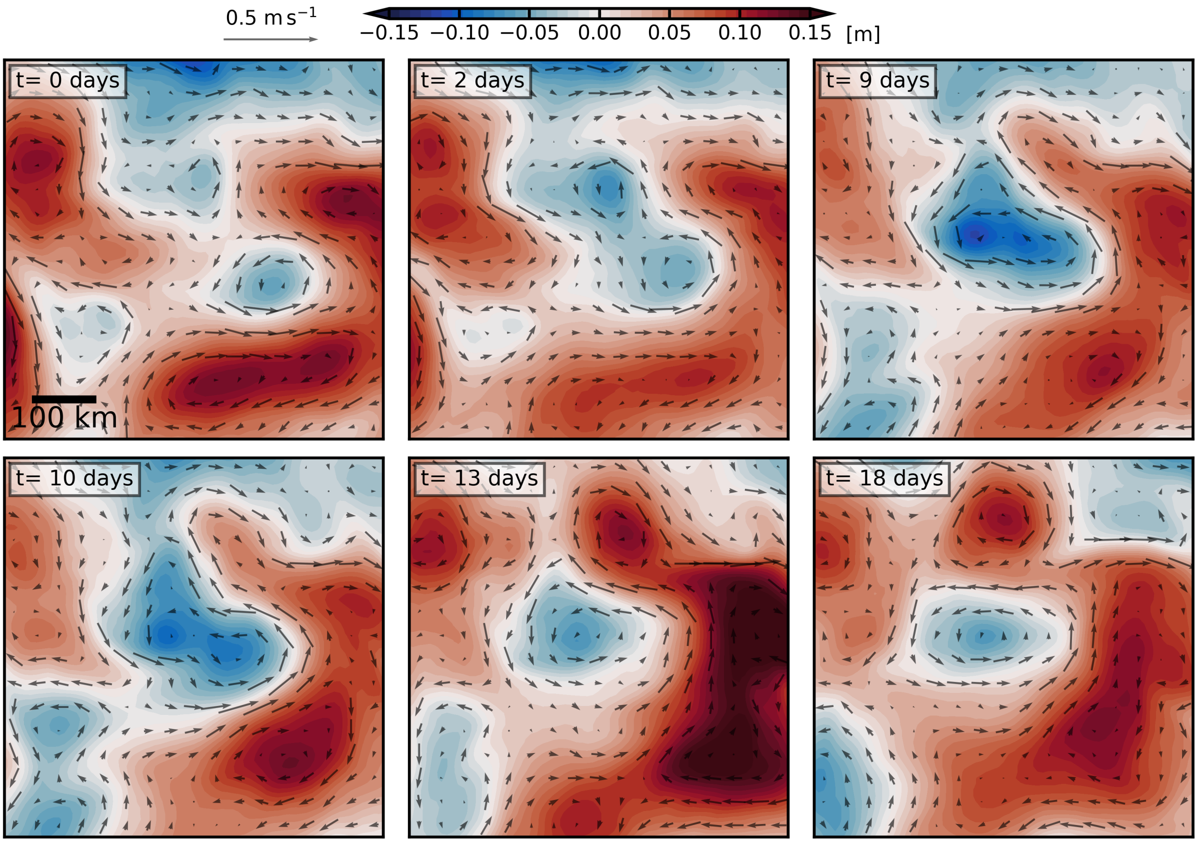

Figure 9: Time evolution of the sea surface height in the HYCOM dataset during the merger of two CEs detected by AMEDA in the North Pacific (NP area). Each panel is centered around 136.48°W, 19.68°N. The first panel shows the sea surface height on 2003/04/10. Black arrows indicate the surface geostrophic velocity computed from the sea surface height anomaly. This example illustrates the impact of neighbouring AEs on the merger.

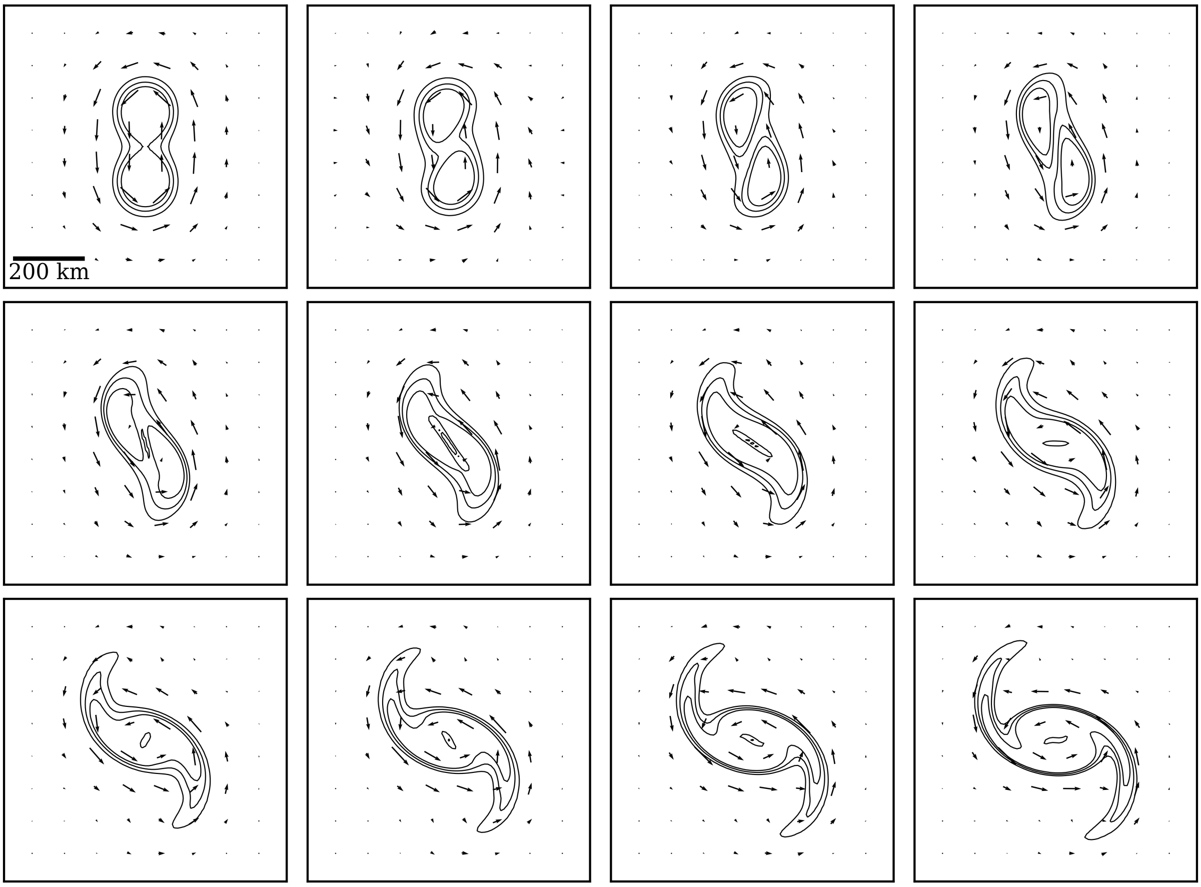

Figure 10: Time evolution of the surface relative vorticity in an idealized 'isolated vortex merging' simulation on the f-plane. The initialization consists of two CEs, with $d/R = 2.5$, $R = 80$ km , $V = 0.3\,\mathrm{m\,s^{-1}}$, $H = 1000$ m, and $\alpha = 2$. Black contours correspond to normalized surface relative vorticity ω_s/f =[0.03,0.05,0.07]; black arrows indicate the surface velocity field. Time goes from left to right and from top to bottom, with a time spacing between panels of 2 days. Note that not the whole domain of the simulation is shown. For this simulation, merger occurs.

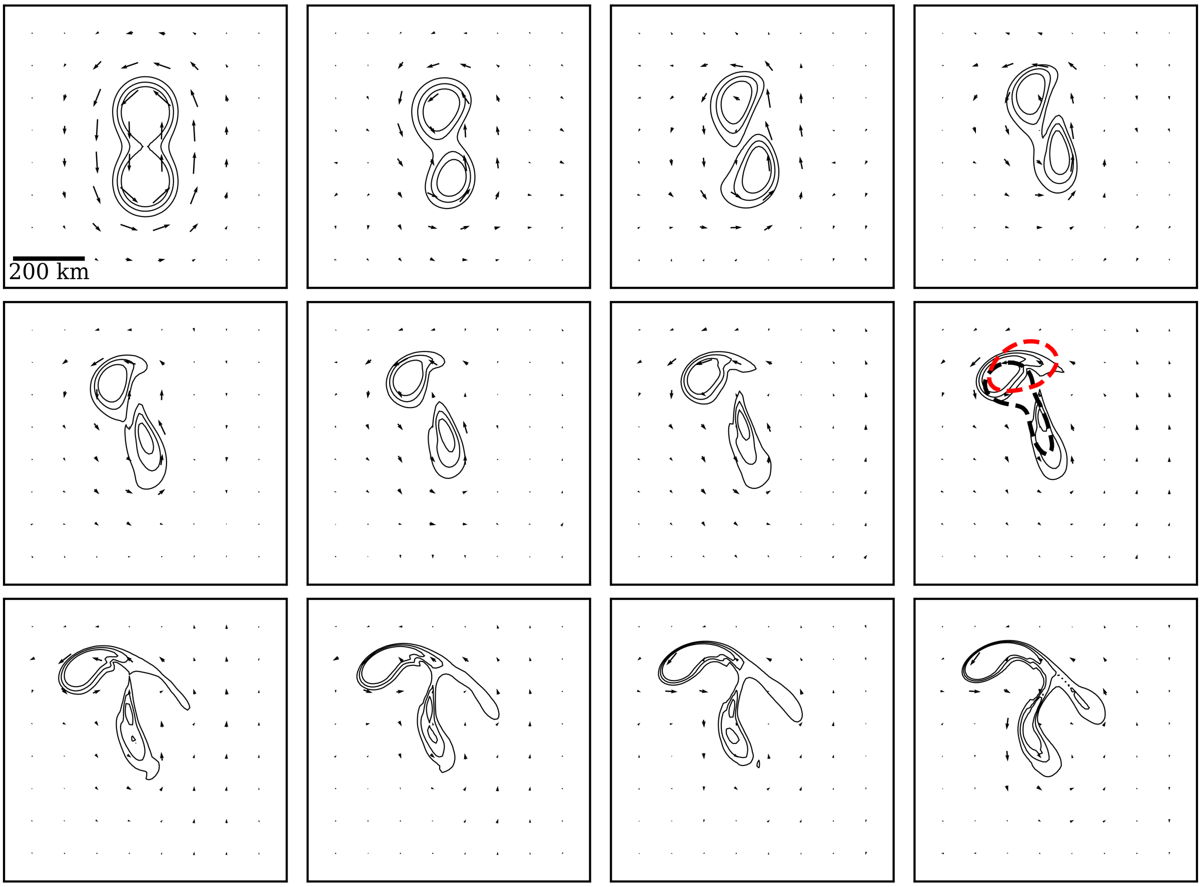

Figure 11: Same as Fig. 10, but for which the simulation was ran on the β-plane. In this case, merger does not occur. The meridionally aligned eddies generate Rossby wave composed of meridionally elongated Sea Surface Height patches with zonally alternating sign (*e.g* Supplementary Fig. 2C). This wave creates a northward velocity east of the two CEs, seen on day 4; thus, the northern eddy moves northward, weakening the merging efficiency. Modifying the orientation of the two eddies at initialization (not shown) tends to slightly reduce this effect. However, even when eddies are zonally aligned, an intense shear generated by Rossby waves leads to the stretching of the two eddies along the zonal axis. This also acts in reducing the merging efficiency. Bold dashed contours in the eighth panels are -0.2 m contours of SSH for the β-plane simulation (black) and the β-disk simulation (red) with the same parameters. For this latter, the merging efficiency is increased and merging is detected.

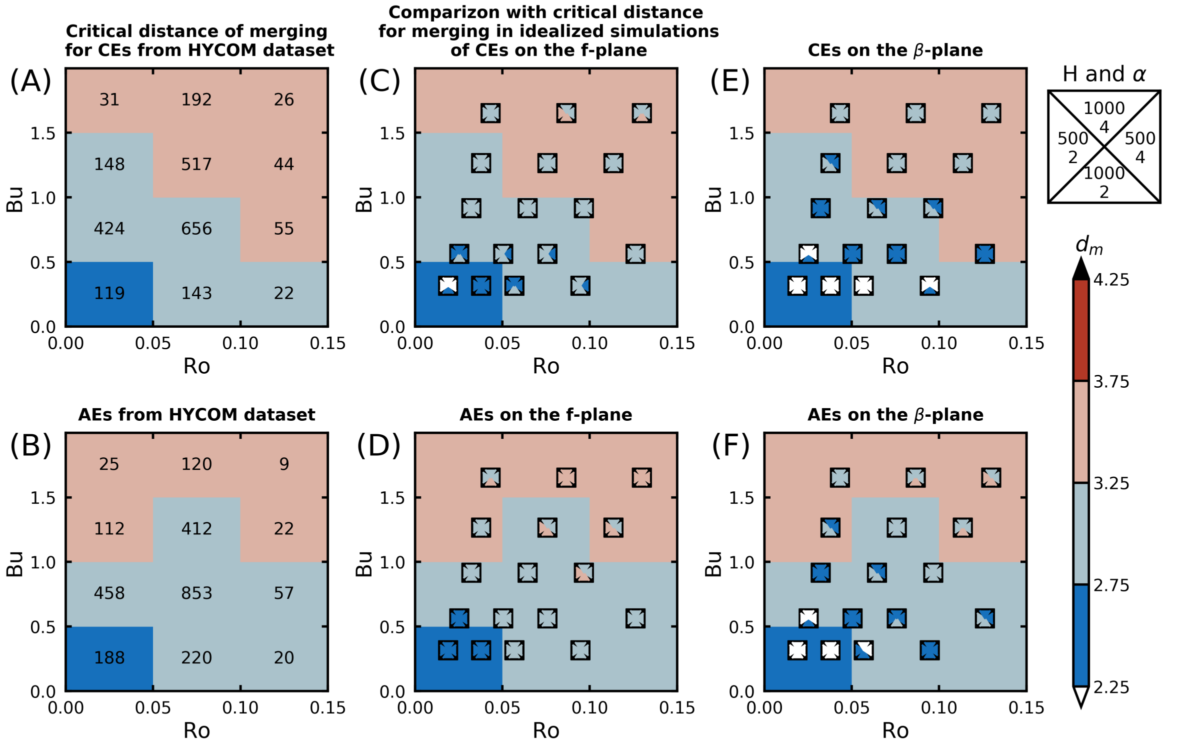

Figure 12: Comparison between the mean distance of merging in the HYCOM dataset, and the maximal initial distance between eddies for which they merge in the idealized simulations. (A) Mean distance of merging d_m for CEs in the HYCOM dataset, depending on Ro and Bu. The numbers of values used to compute averages are indicated for each range of Ro and Bu. (B) Same as (A) for AEs in the HYCOM dataset. (C) Maximum distance of merging d_m^i observed in numerical simulations of CEs on the f-plane. Results are presented as black squares in which 4 grey scale triangles are inserted. Each square corresponds to a given value of Ro and Bu, and the different triangles in this square corresponds to different values of H and α (see top right insert, which indicates the values of H (in meters) and α). The color background is the same as in (A) to allow a comparison between numerical simulations and observations from the HYCOM dataset. (D) Same as (C) for AEs on the f-plane. (E) Same as (C) for CEs on the β-plane.
